# Transbronchial cryobiopsy alone versus combined with traditional forceps biopsy for acute cellular rejection in lung transplant recipients. A diagnostic randomized trial

**DOI:** 10.1016/j.jhlto.2025.100262

**Published:** 2025-04-03

**Authors:** Carolin Steinack, Maurice Roeder, Silvan Vesenbeckh, Martina Haberecker, Jan H. Rüschoff, René Hage, Silvia Ulrich, Malcolm Kohler, Macé M. Schuurmans, Daniel P. Franzen, Thomas Gaisl

**Affiliations:** a Department of Pulmonology, University Hospital Zurich, Zurich, Switzerland; b Department of Pathology and Molecular Pathology, University Hospital Zurich, Zurich, Switzerland; c Department of Internal Medicine, Spital Uster, Uster, Switzerland

**Keywords:** acute cellular rejection, cryobiopsy, forceps biopsy, lung transplant recipients, transbronchial biopsy

## Abstract

**Background:**

Transbronchial lung biopsy is routinely performed to identify acute cellular rejection (ACR) in lung transplant recipients (LTRs). This trial evaluates the clinical value of forceps and cryobiopsies versus cryobiopsies as a standalone diagnostic tool.

**Methods:**

In this randomized trial, LTRs were randomly assigned to receive either 2 cryobiopsies (cryobiopsy group) or a combination of 5 forceps- and 2 cryobiopsies (combined group). The primary outcome was the diagnostic yield to detect ACR; the secondary outcome was the incidence of ACR. We conducted a paired, intraindividual comparison in the combined group alongside interindividual comparisons.

**Results:**

A total of 80 LTRs were randomly assigned to the cryobiopsy group (*n* = 40) or the combined group (*n* = 40) with 90 and 87 procedures performed, respectively. The diagnostic yield for ACR in the cryobiopsy group was similar to the combined group (95.6% vs 97.7%, *p* = 0.430). The sole use of cryobiopsies did not lead to a lower ACR incidence compared to the combined group (10% vs 17.2%, risk ratio 2.21 [95% confidence interval (CI) 0.67-7.29]; *p* = 0.190). Adverse events did not differ between the 2 groups (60.9% vs 57.5%, *p* = 0.655). The pneumothorax rate was overall 1.7%. There were no deaths or occurrences of severe bleeding.

**Conclusions:**

Cryobiopsies did not detect lower ACR than the combined group and can be used as primary and standalone diagnostic tools for histologic assessment of ACR without requiring forceps biopsies.

## Background

Acute cellular rejection (ACR) is a frequent complication within the first year after lung transplantation (LTx) and a significant risk factor among several immune-mediated allograft injuries for chronic allograft dysfunction (CLAD) and mortality.^1^ At least one case of treated ACR has been documented among almost 28% of patients after LTx within the first year.^2^ ACR and CLAD are the leading causes of post-transplant morbidity and mortality, which limit the overall median survival to 5 or 6 years.^2^

Readily accessible diagnostic procedures to detect ACR at the earliest possible occasion are crucial for post-transplant survival. Histopathological confirmation is essential for diagnosing ACR. The current standard for diagnosing and grading ACR, with the consequence of adjusting immunosuppression, is forceps biopsy (FB).^3^ This biopsy method is still limited due to small sample sizes and crush artifacts. Transbronchial lung cryobiopsy (CB), a validated technique for diagnosing interstitial lung disease and lung cancer, can offset the disadvantages of FB by gaining larger specimens with fewer artifacts.^4^, ^5^, ^6^ However, there is still insufficient evidence for CB in the diagnosis and screening of ACR. CB's value in obtaining a conclusive diagnosis of ACR is discussed controversially due to the lack of safety and efficacy data in lung transplant recipients (LTRs).^7^, ^8^, ^9^, ^10^, ^11^, ^12^ In our recent analysis of LTRs, CB, compared with FB, provided improved diagnostic accuracy for ACR with an acceptable safety profile, leading to reclassification and a change of treatment strategy in 23.8% of cases.^13^ However, until today, no randomized controlled trial (RCT) compares CB with FB in bronchoscopies for surveillance purposes. In our current trial, we used our internal standard diagnostic approach of obtaining FB and CB sequentially during the same bronchoscopy and compared it to CB alone. The goal was to establish CB as a standalone method.

## Material and methods

### Patient selection and overall study design

Between July 2021 and December 2023, all adult LTRs undergoing clinically indicated and routinely performed bronchoscopy 2, 4, 6, and 12 months after LTx at the University Hospital Zurich were included in this investigator-initiated, parallel, open-label trial, diagnostic RCT. Participants aged 18 years an older were randomly assigned 1:1 by a computer program to receive either 2 CB (cryobiopsy group) with a 2.4 mm probe alone (freezing time 3-4 seconds) or 5 transbronchial FB and 2 CB (combined group). Patients were excluded if they had any contraindications for bronchoscopy (e.g., comorbidities): international normalized ratio (INR) > 2, thrombocytes <50,000 per microliter of blood, double antiplatelet drugs (e.g., aspirin and clopidogrel) within 7 days before bronchoscopy, oral anticoagulants with nonvitamin K antagonist within 48 hours before biopsy, or relevant pulmonary hypertension (mean pulmonary arterial pressure >30 mm Hg or estimated gradient between the right ventricle and the right atrium >35 mm Hg). LTRs with pulmonary infection or neutropenia were also excluded from the study. Pathologists dedicated to LTx evaluations and blinded to the study arm allocation assessed all biopsies according to International Society of Heart and Lung Transplantation (ISHLT) criteria (A0-A4).^3^ In cases of ACR grade A1, the prednisolone dosage was increased by 10 mg/d for 5 days. In cases of grade A2 rejection, intravenous steroid pulse therapy with methylprednisolone 125 mg/d (1-2 mg/kg/d) for 3 days was given. In cases of persistent grade A2 or A3 rejection, the methylprednisolone pulse dose (10-15 mg/kg/d) was increased to 0.5 or 1 g/d over 3 days, and the calcineurin inhibitor was switched if tacrolimus was not yet administered. All patient-related data, including clinical, demographic, and pathology data, as well as reports of bronchoscopy and spirometry, were obtained from electronic patient records. The ethics committee of the Canton of Zurich approved the study (BASEC-ID 2021-00466), which was registered at clinicaltrials.gov (NCT05006742). The study was consistent with the ISHLT ethical statement. All participants provided written informed consent.

### Bronchoscopy and peri-interventional care

Vitamin K-antagonist anticoagulants were discontinued 5 to 7 days before the procedure. Patients with an INR > 1.3 and ≤2 received vitamin K, so the INR was ≤1.3 on the day of bronchoscopy. Empiric intravenous antibiotic treatment was initiated on the day before bronchoscopy and continued until discharge on the day after bronchoscopy according to or clinical internal protocol. Bronchoscopies were usually conducted in moderate sedation using local anesthesia (lidocaine) and intravenous propofol. Flexible bronchoscopes (Olympus, Tokyo, Japan, 190 series) were inserted through an uncuffed (BronchoFlex, Rüsch, size inner diameter 7.5 mm, Germany) or cuffed tracheal tube (size 7.5-8.5) to prevent laryngeal injury and to maintain a safe airway in case of complications. Bronchoscopy procedure started with a bronchoalveolar lavage with 150 to 200 ml of normal saline followed by 5 specimens obtained by FB and 2 specimens by CB from 2 lobes (combined group) or 2 specimens from 2 lobes by CB (cryobiopsy group) of the same lung side. In the combined group, FB and CB were performed consecutively within the same session, each according to official recommendations, and obtained unilaterally in different segments.^8^, ^14^, ^15^ Fluoroscopy guided the forceps and cryoprobe (Erbe 2.4-mm, Elektromedizin GmbH, Tübingen, Germany), advancing the way to the pleura, and then retracted by 2 to 3 cm. For FB, anterior and posterior segments of the lower lobes were preferred. CB was preferably obtained from the laterobasal bronchus and one of the segmental bronchi of the upper lobe, a total of 5 specimens by FB and another 2 by CB. When available, the location of biopsies was independent of possible CT findings. A freezing time of 3 to 4 seconds with the 2.4 mm cryoprobe was applied; thereafter, the cryoprobe and bronchoscope were removed en bloc. Xylometazoline (2 mg) and tranexamic acid (500 mg) were prepared to be instilled over the bronchoscope's working channel in the case of moderate to severe bleeding. In addition, a bronchial blocker (Rüsch, size Ch.6) was always ready to be used but not prophylactically installed. After thawing the CB sample in normal saline at room temperature, it was directly transferred to a formalin solution. FB samples were directly put into the formalin solution. Every procedure was performed by respiratory physicians experienced in CB, with a minimum of 30 procedures per year. Pneumothorax was screened with chest radiography in an upright position 2 to 4 hours after the procedure. Bleeding grade was scored according to Nashville bleeding score.^16^ Grade 1 bleeding requires the suctioning of blood for <1 minute, whereas grade 2 bleeding requires suctioning for more than 1 minute and repeated wedging or instillation of vasoactive substances or thrombin, respectively. The premature interruption of the procedure, balloon blocker insertion for <20 minutes, or selective intubation is classified as grade 3 bleeding. Grade 4 bleeding is defined as selective intubation or balloon blocker insertion for longer than 20 minutes in addition to red blood cell transfusion, selective bronchial artery embolization, admission to the intensive care unit, surgical intervention, or resuscitation.

### Data analysis

The primary outcome was the difference in diagnostic yield defined as suitability of all specimens to detect ACR according to the ISHLT histopathological criteria in each arm (cryobiopsy group vs combined group).^3^ The secondary outcome was the incidence of ACR during the trial.

Continuous outcomes were summarized using mean ± standard deviation or median (interquartile range) as appropriate. The prespecified analyses used data from the full-analysis set, which included all patients who underwent randomization and received at least 1 biopsy. The safety analysis set included all patients randomly assigned to a study group and had a biopsy. No missing values were imputed. Prespecified intraindividual diagnostic analyses included participants who had both CB and FB, which allowed for comparing the 2 sampling strategies.

The primary outcome was analyzed using multilevel mixed-effects logistic regression without weights to compare dichotomous variables between groups. Furthermore, we conducted a paired, intraindividual comparison in the combined group (CB and FB). The denominators of the diagnostic yield analyses were calculated based on the number of patients who had the biopsy (primary analysis).

Categorical data were compared using Pearson’s chi-square or Fisher’s exact test as appropriate, and inference for stratified categorical data. For continuous data, between-group comparisons were performed by Student’s *t*-test for parametric variables or the Mann-Whitney U test for nonparametric variables. For statistical significance, a 2-sided *p*-value of <0.05 was used. All analyses were conducted using STATA version 18.0 (StataCorp LP, College Station, TX).

## Results

### Baseline characteristics

Between July 1, 2021, and December 27, 2023, 82 patients were assessed for eligibility. A total of 80 patients were enrolled and randomly assigned to either the combined group (*n* = 40) or the cryogroup (*n* = 40) with 87 and 90 procedures, respectively. Two patients were excluded because one had a pulmonary infection, and one had an abnormal INR >2. None of the patients declined participation in the study. All biopsies (CB and FB) were completed according to the study protocol; each LTR had on average, 2.2 bronchoscopies, with a minimum of 1 and a maximum of 6. Not all 4 procedures mentioned in our protocol could be performed within the first year following LTx due to different reasons (pulmonary infections, neutropenia, LTR preference, later time of first biopsy due to frailty), and not all procedures of each LTR were within the time-frame of this study. The study groups' baseline characteristics were well-balanced (Table 1). A total of 49 (61.3%) of 80 participants were male, and the mean age was 56.6 ± 10 years. All LTRs had lifelong triple immunosuppression consisting of a corticosteroid, a calcineurin inhibitor (tacrolimus or cyclosporin), and an antimetabolite (mycophenolate mofetil) or mammalian target of rapamycin (mTOR) inhibitor (everolimus).

**Table 1:** Baseline Characteristics of the Patients

	Cryobiopsy only	Cryobiopsy + Forceps	*p* value
Lung transplant recipients—*n* (%)	40 (50)	40 (50)	
Age, year ±SD	57.1 ± 9.2	56.1 ± 10.7	
Sex—*n* (%)			
Female	13 (32.5)	18 (45.0)	
Male	27 (67.5)	22 (55.0)	
Disease—*n* (%)			
COPD	21 (52.5)	25 (62.5)	
Sarcoidosis	0 (0.0)	2 (5.0)	
Interstitial lung disease	13 (32.5)	6 (15.0)	
Cystic fibrosis	2 (5.0)	5 (12.5)	
Pulmonary hypertension	0 (0.0)	2 (5.0)	
Sjögren Syndrome	1 (2.5)	0 (0.0)	
Bronchiolitis	2 (5.0)	0 (0.0)	
Pulmonary venoocclusive disease	1 (2.5)	0 (0.0)	
Side of lung transplantation—*n* (%)			
Bilateral	36 (90.0)	36 (90.0)	
Unilateral	4 (10.0)	4 (10.0)	
Air trapping—*n* (%)	13 (32.5)	13 (32.5)	
Immunosuppression			
Prednisone, mg	13 [8-15]	11 [8-19]	
Tacrolimus	14 (35.0)	15 (37.5)	
Tacrolimus, mg	1.0 [0.8-2.2]	1.0 [0.6-2.0]	
Ciclosporin	26 (65)	23 (57.5)	
Ciclosporin, mg	120 [80-170]	120 [80-160]	
Mycophenolic acid	24 (60)	25 (63)	
Mycophenolic acid, mg	2,500 [1,500-3,000]	3,000 [2,500-3,500]	
Everolimus	3 (8)	6 (15)	
Everolimus, mg	0.4 [0.3-0.5]	0.5 [0.3-0.5]	
Mycophenolate mofetil	12 (30)	10 (25)	
Mycophenolate mofetil, mg	2,160 [1,440-2,160]	1,800 [720-2,160]	
Number of procedures—*n* (%)	90 (50.8)	87 (49.2)	
Days since lung transplantation, d	224 [132-364]	233 [136-380]	0.696
Side of biopsy—*n* (%)			0.841
Right	51 (56.7)	48 (55.2)	
Left	39 (43.3)	39 (44.8)	
Air trapping—*n* (%)	13 (32.5)	13 (35.1)	
Indication bronchoscopy—*n* (%)	27 (30.0)	24 (27.6)	0.723
Oral anticoagulation—*n* (%)	18 (20.0)	9 (10.3)	
Platelet aggregation inhibitors—*n* (%)	12 (13.3)	13 (15.1)	
Hemoglobin, g/liter	111 [102-123]	112 [98-123]	
Leukocytes, g/liter	6.3 [4.4-9.5]	6.020 [4.7-8.2]	
Lymphocytes, g/liter	0.6 [0.4-0.9]	0.7 [0.4-1.1]	
Thrombocytes, g/liter	240 [199-295]	252 [209-314]	
INR	1.0 [0.9-1.0]	1.0 [0.9-1.0]	
C-reactive protein, mg/liter	1.0 [0.6-2.5]	1.0 [0.6-2.3]	
FEV1, liters	2.2 [1.8-2.9]	2.5 [1.8-3.2]	
DLCO, %pred.	56 [50-73]	67 [56-76]	

Abbreviations: COPD, chronic obstructive pulmonary disease; DLCO, diffusing capacity for carbon monoxide; FEV1, forced expiratory volume in the first second; INR, international normalized ratio; SD, standard deviation.

Plus-minus values are mean ± standard deviation. Values in square brackets are median [interquartile range].

Air trapping is detected on chest computed tomography by the depiction of hyperinflated lung areas due to abnormal air retention, and may be observed in patients with chronic lung allograft dysfunction.

### Outcomes

The overall diagnostic yield according to the ISHLT histopathological criteria was 86 (95.6%) out of 90 procedures in the cryobiopsy group and 85 (97.7%) out of 87 procedures in the combined group (*p* = 0.430). The sole use of a CB did not lead to a significantly lower ACR incidence in the cryobiopsy group compared to the combined group (9 [10%] out of 90 procedures versus 15 (17.2%) out of 87 procedures, risk ratio [RR] 2.21 [95% confidence interval (CI) 0.67-7.29]; *p* = 0.190). Specimen qualities were similar between the 2 groups regarding evaluable lung specimens (Table 2). In 6 procedures, there was suspicion of antibody-mediated rejection, but C4d staining in the CB and FB was negative. Three patients with obliterative bronchiolitis, one with organizing pneumonia, and 2 with histologically proven CMV pneumonitis were only diagnosed with CB specimens.

**Table 2:** Diagnostic Yield, Specimen Properties, and Adverse Events in the Entire Cohort (Interindividual Comparisons)

	Cryobiopsy alone	Forceps and cryobiopsy	Total	*p* value
Procedures	90	87	176	
ACR diagnosis	9 (10)	15 (17.2)	24 (13.6)	0.190
Grade of ACR				0.124
A0	81 (90)	72 (82.8)	153 (86.4)	
A1	5 (5.6)	4 (4.6)	9 (5.1)	
A2	3 (3.3)	11 (12.6)	14 (7.9)	
A3	1 (1.1)	0 (0.0)	1 (0.6)	
B0	0 (0.0)	0 (0.0)	0 (0.0)	
B1	0 (0.0)	0 (0.0)	0 (0.0)	
B2	0 (0.0)	0 (0.0)	0 (0.0)	
B3	0 (0.0)	0 (0.0)	0 (0.0)	
Quality of biopsies (A)				0.430
Sufficient (ISHLT)	86 (95.6)	85 (97.7)	171 (96.6)	
Insufficient (ISHLT)	4 (4.4)	2 (2.3)	6 (3.3)	
Quality of biopsies (B)				0.182
Sufficient (ISHLT)	79 (87.8)	70 (80.5)	149 (84.2)	
Insufficient (ISHLT)	11 (12.2)	17 (19.5)	28 (15.5)	
Days since lung transplantation	224 (132-364)	233 (136-380)	224 (134-376)	0.696
Indication bronchoscopy, *n* (%) yes	27 (30.0)	24 (27.6)	51 (28.8)	0.723
Side of the biopsy				0.841
Right—*n* (%)	51 (56.7)	48 (55.2)	99 (55.9)	
Left—*n* (%)	39 (43.3)	39 (44.8)	78 (44.1)	
Size of cryobiopsies, mm	7 (6-10)	7 (5-10)	7 (5-10)	
Size of forceps biopsies, mm	-	2 (1-3)	-	
				
Adverse events—*n* (%)				
				
Pneumothorax—Grade 1	0 (0)	0 (0)	0 (0)	-
Pneumothorax—Grade 2	1 (1)	0	1 (1)	-
Pneumothorax—Grade 3	0 (0)	0 (0)	0 (0)	-
Bleeding—Grade 0	39 (43.3)	34 (39.1)	73 (41.2)	0.568
Bleeding—Grade 1	16 (17.8)	17 (19.5)	33 (18.6)	0.865
Bleeding—Grade 2	27 (30.0)	29 (33.3)	56 (31.6)	0.684
Bleeding—Grade 3	8 (8.9)	7 (8.1)	15 (8.5)	0.538
Bleeding—Grade 4	0 (0)	0 (0)	0 (0)	-
Dyspnea				-
Death	0 (0)	0 (0)	0 (0)	-

Abbreviation: ACR, acute cellular rejection.

Pneumothorax grading according to British Thoracic Society guidelines. Bleeding grading according to Nashville classification.

In a paired subgroup analysis of the combined group (*n* = 40), CB led to a higher ACR incidence compared to FB (15 [17.2%] out of 87 biopsies, vs 2 [2.3%] out of 87 biopsies, RR 5.12 [95% CI 3.30-7.93]; *p* = 0.001). The suitability for ACR diagnosis according to ISHLT criteria of the CB and the FB is statistically significantly different (90.8% vs 19.5%, *p* < 0.001) (Table 3).

**Table 3:** Comparison of Cryobiopsies and Forceps Biopsies in the Combined Group Based on 87 Procedures in 40 Individual LTRs

	Cryobiopsy (*n* = 87)	Forceps biopsy (*n* = 87)	*p* value
ACR diagnosis	15 (17.2)	2 (2.3)	0.003
Grade of ACR			0.003
A0	66 (75.9)	79 (90.8)	
A1	4 (4.6)	1 (1.1)	
A2	11 (12.6)	1 (1.1)	
B0	0 (0.0)	0 (0.0)	
B1	0 (0.0)	0 (0.0)	
B2	0 (0.0)	0 (0.0)	
Quality of biopsies (A)			<0.001
Sufficient (ISHLT)	79 (90.8)	17 (19.5)	
Insufficient (ISHLT)	2 (2.3)	64 (73.6)	
Quality of biopsies (B)			0.436
Sufficient (ISHLT)	74 (85)	66 (76)	
Insufficient (ISHLT)	13 (15)	21 (24)	
Number of sufficient probes available			<0.001
1	4 (4.4)	0 (0)	
2	78 (86.7)	0 (0)	
3	8 (8.9)	18 (20.7)	
4	0 (0)	23 (26.4)	
5	0 (0)	42 (48.3)	
6	0 (0)	3 (3.5)	
7	0 (0)	1 (1)	
Size of biopsies, mm	7 (5-10)	2 (1-3)	<0.001

Abbreviation: ACR, acute cellular rejection.

For the cryobiopsy group, a median of 2 (IQR 2-2) CB was obtained, and for the combined group, a median of 5 (IQR 4-5) FB and a median of 2 (2-2) CB were obtained. The size of the CB had a median of 7 mm (IQR 5-10), while the FB had a median of 2 mm (IQR 1-3).

Out of 177 procedures, 3 cases (1.7%) resulted in pneumothoraces that resolved within 48 hours without requiring a chest tube insertion. The adjusted pneumothorax rate (accounting for the multiple biopsy methods) was 1.1%. Additionally, we observed several instances of mild (grade 1 bleeding 18.6%) and moderate bleeding (grade 2 bleeding 31.6%), but no grade 4 bleeding (defined as a new admission to the intensive care unit, resuscitation, red blood cell transfusion or selective bronchial artery embolization) without significant differences between the cryobiopsy group and combined group. Due to persistent bleeding during bronchoscopy, 8.5% of patients required a bronchial balloon (grade 3), which could be removed after a few minutes during the same procedure (Table 2). Bleeding grade did not correlate with the use of aspirin, warfarin, and apixaban, which were used in 16, 12, and 2 procedures, respectively. There was also no association between the days since LTx and ACR grade (*p* = 0.353).

### Follow-up

All patients had follow-up appointments 1 or 2 weeks after hospital discharge after bronchoscopy. Two patients had extended oral antibiotic treatment for up to 14 days to prevent pneumonia due to ongoing mild hemoptysis.

## Discussion

This diagnostic RCT involving 80 LTRs with 177 procedures suggests that transbronchial CBs can be used as a standalone diagnostic tool to detect ACR (Table 2). The trial showed that CBs led to a higher ACR incidence compared to FBs with an RR of 5.12 (95% CI 3.30-7.93); *p* = 0.001 (Table 3). The latter should be interpreted with caution, since only 18.7% of the FB in 5 forceps sample met the ISHLT criteria.

The greatest benefit of CB is its larger specimen size, allowing for more precise diagnostics in LTRs.^7^, ^9^ We recently reported that, in 23.8% of patients who underwent both consecutive CB and FB during the same procedure, ACR would have been missed if only FB had been performed.^13^ Consistent with our current analysis, in the combined group (*n* = 40), CB led to a higher ACR incidence compared to FB (15 [17.2%] out of 87 biopsies, vs 2 [2.3%] out of 87 biopsies, RR 5.12 [95% CI 3.30-7.93]; *p* = 0.001).

We chose the 2.4 mm cryoprobe as outlined in our study protocol due to its proven effectiveness, delivering sufficiently large lung specimens in 95.6% of cases when used alone. Compared to the 1.9 mm cryoprobe and forceps, the 2.4 mm probe consistently provided superior diagnostic yield and specimen quality.^17^ Its ability to obtain larger tissue samples made it the optimal choice for accurate diagnosis and analysis. Additionally, CB offers the advantage of shorter fluoroscopy and procedure times compared to FB, as fewer CB specimens are needed to achieve a sufficient diagnosis.^9^ While we did not measure our procedure time, it was observed that overall procedures were faster in the cryobiopsy group, further supporting its efficiency.

Considering the advantages of obtaining larger, higher-quality specimens with fewer artifacts, it is essential to weigh these benefits against the potential risks of CB. In a cohort study involving 402 procedures, CB and FB, when performed at different time points, demonstrated no significant differences in the risks of pneumothorax and bleeding. This finding suggests that, in centers with CB experience, the benefits of CB in producing superior specimens can be achieved without increasing procedural risks, making it a favorable option in clinical practice,^10^ which is supported by our data. The overall pneumothorax rate remained low at 1.7% despite the sequential use of FB and CB during the same procedure in the combined group, compared to the pneumothorax rate of 1% to 13% reported in the literature.^7^, ^9^, ^10^, ^11^, ^12^, ^18^, ^19^ Mild and moderate bleeding, which can be easily managed by an interventional pulmonologist, was low, with 18.6% grade 1% and 31.6% grade 2 bleeding. Authors use different definitions of bleeding grades in the literature. Thus, it is difficult due to the lack of a uniform used definition of the degree of bleeding. Loor et al^19^ observed a bleeding grade 3 (use of a bronchial blocker) in 4.6% (*n* = 1) of the patients. The lower incidence of grade 3 bleeding can be attributed to the significantly smaller number of total procedures (*n* = 22) compared to the 8.5% (*n* = 15) of grade 3 bleeding cases out of 177 procedures in our cohort.^19^ Nevertheless, bleeding events occurred more frequently when the cryoprobe, particularly the 2.4 mm probe was used compared to the cryoprobe 1.9 mm and forceps.^17^ Consequently, we suggest that experienced interventional pulmonologists carry out CB for LTRs, as not many pulmonologists are familiar with placing a tracheal tube or a bronchial blocker. For safety reasons, all CB require subglottic airway management with a tracheal tube or rigid tube. We opted for a tracheal tube with propofol, which does not require an anesthesiologist in our country. We are aware that this is not the norm in all countries.^20^

The secondary outcome of this study was the incidence of ACR. This was chosen because questions have been raised in recent years concerning the value of scheduled surveillance bronchoscopies in asymptomatic LTRs.^21^, ^22^ McWilliams et al^22^ detected similar ACR grades ≥2 in surveillance and bronchoscopies with clinical indication (18.7% vs 15.7%). The group of Mohamed et al^12^ mentioned ACR A1 in 44%, A2 in 8%, and A3 in 1% of LTRs who underwent bronchoscopy for surveillance purposes. 126 out of 177 procedures were scheduled for surveillance reasons in our analysis, in which we detected 5.1% A1, 7.9% A2, and 0.6% A3 ACR, highlighting the clinical profit of surveillance bronchoscopies, and keeping in mind that the main goal is to treat ACR in early stages before lung function declines and CLAD develops. Furthermore, the pace of tapering the triple immunosuppression from a maximal level early post transplant to maintenance immunosuppression at about 1 year after LTx may be influenced when ACR is excluded.

Our study has several limitations. First, it is based on a single-center analysis, which may limit the generalizability of the findings. Second, the pathologists were not blinded in the combined group but were asked to assess the FB specimens first, followed by the CB specimens. Third, the optimal number of specimens needed is unclear. However, evidence suggests that 2 CB specimens are generally sufficient for achieving an accurate ACR diagnosis.^13^ Obtaining 5 specimens with forceps according to our study protocol could not provide acceptable diagnostic yield according to the ISHLT histopathological criteria, as compared to CB (18.7% vs 97.3%, *p* = 0.003), underestimating the value of FB in our study. Therefore, we recommend collecting more than 5 FB upon the bronchoscopist’s decision to aim well evaluable specimens according to ISHLT criteria.^3^, ^9^, ^10^ Since we started with FB followed by CB in the combined group, bleeding was observed at the end of the procedure after obtaining CB. Therefore, bleeding complications can be attributed to CB, which is a limitation of this design. Another limitation is the 2-night in-hospital stay required by our study protocol, as this practice is likely uncommon in high-volume transplant centers.

The greatest strength of our trial lies in its being the first diagnostic RCT in LTRs to evaluate the effectiveness of standalone CB for detecting ACR during both surveillance bronchoscopies and those performed for clinical indications. Unlike most previous studies, which rely on historical comparisons with FB, our study provides direct, contemporaneous evidence of CB's value in this specific patient population.^9^, ^10^, ^12^ Furthermore, our study encompasses a high number of procedures, with 44 patients undergoing 2 bronchoscopies, 51 undergoing 3, 32 undergoing 4, 15 undergoing 5, and 6 undergoing 6 bronchoscopies.

**Figure 1:**
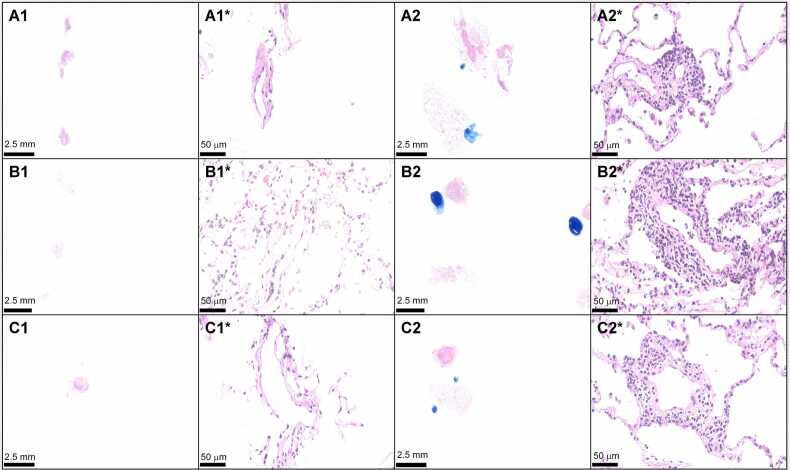
Histologic transbronchial biopsy specimens of 3 patients (A, B, C). Forceps biopsies (A1, A1*, B1, B1*, C1, C1*) are smaller and contain less alveoli and small airways than cryobiopsies (A2, A2*, B2, B2*, C2, C2*). Acute cellular rejection grade 2 was detected exclusively in cryobiopsies (A2, A2*, B2, B2*, C2, C2*) in all 3 patients. (hematoxylin-eosin).

In conclusion, based on our results, we advocate for CB as the primary and standalone diagnostic tool for histologic assessment of ACR in LTRs. However, long-term follow-up studies are essential to fully establish the safety profile of CB in this patient population (Figure 1).

## Ethics statement

The study was performed in the Department of Pulmonology at the University Hospital Zurich, Zurich, Switzerland, and approved by the Cantonal Ethics Committee in Zurich, Switzerland (ID-2021-00466).

## Author contributions

C. Steinack and T. Gaisl had full access to all data in the study and took responsibility for the integrity of the data and the accuracy of the data analysis. Study concept and design: C. Steinack, D.P. Franzen. Acquisition of data: C. Steinack, T. Gaisl, M. Roeder, S.M. Vesenbeckh, J.H. Rüschoff, M. Haberecker, D.P. Franzen. Analysis or interpretation: C. Steinack, H. Rüschoff, S. Ulrich, M. Haberecker, T. Gaisl, D.P. Franzen. Drafting of the manuscript: C. Steinack, T. Gaisl. Critical revision of the manuscript for important intellectual content: All authors. Statistical analysis: T. Gaisl. Administrative, technical, or material support: C. Steinack, J.H. Rüschoff, M. Haberecker, T. Gaisl. Study supervision: M. M. Schuurmans.

## Disclosure statement

M. Kohler received consulting fees from Novartis. He is cofounder and shareholder of Deep Breath Intelligence AG, a company that provides services in the field of breath analysis. D.P. Franzen received speaker fees from ERBE. None of the other authors declared conflicts of interest.

We sincerely thank the lung transplant recipients who graciously agreed to participate in this study. Their trust and commitment were invaluable, making this analysis possible.

The study has no funding.

## Data Availability

The data supporting this study's findings are available upon reasonable request from the corresponding author.
