# Supplementary material for: Transbronchial cryobiopsy alone versus combined with traditional forceps biopsy for acute cellular rejection in lung transplant recipients. A diagnostic randomized trial
Source: JHLT Open. 2025 Apr 3;8:100262. doi: 10.1016/j.jhlto.2025.100262 (PMC12142631; doi:10.1016/j.jhlto.2025.100262)
Supplement: Supplementary file 1 — Supplementary material [file mmc1.docx]

**Tweet**

Transbronchial cryobiopsies detect more acute cellular rejections than forceps biopsies and can be used as primary and standalone diagnostic tool for histological assessment of acute cellular rejection in lung transplants.
